# PCR Cycle Threshold–Guided Management of Pediatric *Clostridioides difficile* Infections

**DOI:** 10.3390/microorganisms14020313

**Published:** 2026-01-29

**Authors:** Mohammed Suleiman, Andrés Pérez-López, Neama Esmat Salieh

**Affiliations:** 1 Department of Pathology, Sidra Medicine, Doha P.O. Box 26999, Qatar; aperezlopez@sidra.org (A.P.-L.); nsalieh@sidra.org (N.E.S.); 2 Department of Biomedical Sciences, Qatar University, Doha P.O. Box 2713, Qatar; 3 Department of Pathology and Laboratory Medicine, Weill Cornell Medicine in Qatar, Doha P.O. Box 24144, Qatar

**Keywords:** *Clostridioides difficile*, Ct value, pediatric, intervention

## Abstract

This follow-up study expands on our previous work demonstrating that a PCR cycle threshold (Ct)–guided diagnostic approach improves the management of pediatric *Clostridioides difficile* infection (CDI) and reduces unnecessary treatment of colonized children. We evaluated the performance of the Gastroenteritis PCR Panel by QIAstat-Dx as a standalone method in combination with the PCR cycle threshold (Ct) value in PCR-positive samples to predict the presence of free toxins. In addition, we evaluated the impact of reporting toxin production results based on PCR Ct value alongside a comment in our electronic medical record. The QIAstat-Dx assay achieved 100% sensitivity and negative predictive value (NPV), with a specificity of 69% and a positive predictive value (PPV) of 63%. When 16 false-positive samples that were co-infected with other enteropathogens were excluded, the specificity increased to 97%. We observed a significant decrease (51% vs. 68%) in the proportion of treated patients in this study compared to the pre-intervention period of our previous study (*p* = 0.04). In contrast, a minor, non-significant 5% increase (*p* = 0.60) was observed in this study compared with the post-intervention period (45% treated) from the previous study. These findings demonstrate that Ct-guided diagnostic strategies continue to enhance *C. difficile* diagnostic precision and help limit inappropriate antibiotic use in our pediatric population.

## 1. Introduction

*Clostridioides difficile* remains a major cause of healthcare associated diarrhea and has been recently associated with community-acquired diarrhea in children, with clinical consequences ranging from prolonged hospitalization to increased healthcare costs [1]. Despite its clinical and public health importance, the laboratory diagnosis and management of *C. difficile* infection (CDI) in the pediatric population remains challenging as many children carry toxigenic or non-toxigenic strains without symptoms, complicating the interpretation of positive results [2]. Nucleic acid amplification tests (NAATs) offer superior sensitivity compared to traditional diagnostic methods, such as glutamate dehydrogenase (GDH) and toxin enzyme immunoassays (EIAs). However, since NAATs primarily detect the presence of the toxin genes (*tcdA* and *tcdB*), these assays lack sufficient specificity to discriminate between CDI and asymptomatic colonization, a limitation that represents a significant clinical decision-making challenge [3,4,5]. There is a notable scarcity of studies in the literature on diagnosis and the clinical management of *C. difficile* in children [6,7], which necessitates research for better diagnostic tools to improve the diagnosis of CDI in children.

The QIAGEN QIAstat-Dx gastrointestinal panel (QGP; Hilden, Germany) is a fully automated qPCR multiplex syndromic assay that provides both qualitative results and quantitative Cycle threshold (Ct) values for *C. difficile* and 22 other gastrointestinal pathogens [8]. Ct values have been correlated with bacterial load and may assist in distinguishing between colonization and symptomatic infection [9]. Several studies have shown that lower Ct values reflect higher target gene concentrations and are associated with increased CDI severity and adverse clinical outcomes, highlighting their potential role in supporting clinical management of CDI [7,9,10,11]. However, the limited number of studies conducted in pediatric populations underscores the need for further investigation to validate these findings. In our earlier work, we incorporated Ct value data from the GeneXpert and QIAstat-Dx platforms into the diagnostic pathway alongside clinical findings [12]. Both assays showed equally good performance in our previous study [12]. This approach not only improved diagnostic accuracy but also reduced unnecessary antibiotic use [12]. Building on these findings, a Ct value-guided diagnostic protocol was introduced in our laboratory in 2024 using only the QIAstat-Dx platform. The present study evaluates the performance and clinical impact of this protocol over an 11-month period, focusing on antibiotic utilization and diagnostic accuracy. The aim is to determine whether Ct value-based decision-making can further enhance pediatric CDI management and contribute to antimicrobial stewardship efforts.

## 2. Materials and Methods

### 2.1. Ethical Approval

The Sidra Medicine Institutional Review Board (2042490) approved this study.

### 2.2. Study Design, Setting, and Data Collection

This retrospective observational study was conducted in the Microbiology laboratory at Sidra Medicine, a pediatric tertiary care hospital in the State of Qatar. Our Microbiology laboratory performs *C. difficile* testing using only the QGP assay. This study included all stool samples that tested positive for *C. difficile* toxin A and/or toxin B genes using QGP, collected from pediatric patients (aged 2–18 years old) and submitted for QGP testing between 1 February and 31 December 2024. Our exclusion criteria included formed stool, any repeat sample submitted within 7 days, repeat positive samples from the same patient within 30 days, and samples without sufficient clinical data.

### 2.3. Clinical Review

Patients’ electronic medical records were reviewed for age, gender, ordering unit (Emergency Department, Outpatient Clinics, and inpatient units), underlying medical conditions, clinical presentation, previous antibiotic treatments, gastrointestinal co-infections, and CDI treatment. In the clinical review, a true CDI case (true positive [TP]) was defined based on the clinical criteria established by the Infectious Diseases Society of America (IDSA) and the Society for Healthcare Epidemiology of America (SHEA) [6]. True CDI was defined as three or more loose stools per 24 h with no alternative diagnosis and at least one risk factor (e.g., recent antibiotics, immunosuppression, malignancy, transplant, or inflammatory bowel disease [IBD]) [6].

### 2.4. C. difficile Laboratory Diagnosis: Use of PCR Ct Value

Stool samples were analyzed for Toxin A and Toxin B genes using QIAstat-Dx Gastroenteritis PCR Panel (QGP; Hilden, Germany), and all positive targets were recorded along with their Ct values. Samples were analyzed exclusively using QGP, with no rapid phenotypic assays performed. The assay was performed, and the results interpreted according to the manufacturer’s instructions. The toxin production results based on the Ct values from the QIAstat-Dx PCR panel were reported in the electronic medical record (EMR). PCR-positive samples with a Ct value of 27.2 or higher were reported as toxin production negative, while PCR-positive samples with a Ct value of 27.1 or less were reported as toxin production positive. The results were reported along with a comment in the EMR encouraging physicians to correlate the results with the clinical symptoms before starting therapy if the patient was PCR-positive but negative for toxin production. In contrast to our previous study, a follow-up phone call consultation by the infectious disease physician was not performed.

### 2.5. Statistical Analysis

Data were analyzed using OpenEpi Menu (https://www.openepi.com/Menu/OE_Menu.htm, accessed on 7 November 2025 ). A Ct cutoff value of 27.2 was determined using the Youden maximum index while fixing the sensitivity at ≥99%. Similarly to our previous study, clinical review was applied as a reference method [12]. Sensitivity, specificity, positive predictive value (PPV), and negative predictive value (NPV) were calculated with 95% confidence intervals. Chi-square tests were used to evaluate antibiotic prescribing before and after the Ct-guided approach, and *p*-values of <0.05 were considered significant. The results from this study were compared against the published results from our previous study [12].

## 3. Results

### 3.1. Patient Demographics and Clinical Details

Between 1 February and 31 December 2024, a total of 83 patients with PCR-positive *C. difficile* stool samples were identified. The median age of the patients was 7 years (Interquartile range (IQR) 4–11). In total, 37 (45%) of the patients were male, and 46 (55%) were female. Fifty (60%) samples were collected in inpatient units, 18 (22%) in outpatient clinics, and 15 (18%) in the emergency room. Forty-two patients (50.6%) received CDI treatment: 39 (47%) oral vancomycin and 3 (3.6%) oral metronidazole.

### 3.2. Use of PCR Ct Value—Diagnostic Performance

The median Ct value was 25.8 (IQR 23.6–30.4). According to the clinical review, 28 (33.7%) patients were considered to have a true CDI. At a Ct cutoff value of 27.2, the QIAstat-Dx demonstrated 100% sensitivity and NPV, (69%) specificity, and (63%) PPV (Table 1). Sixteen patients who were classified as having false-positive (FP) results (Ct value < 27.2) had an alternative etiology confirmed by a positive PCR for enteropathogens other than *C. difficile*. Performance of the QIAstat-Dx, excluding these 16 FP, is shown in Table 1. One additional patient was classified as FP, as he did not have diarrhea.

### 3.3. Antibiotic Treatment

**Figure 1:**
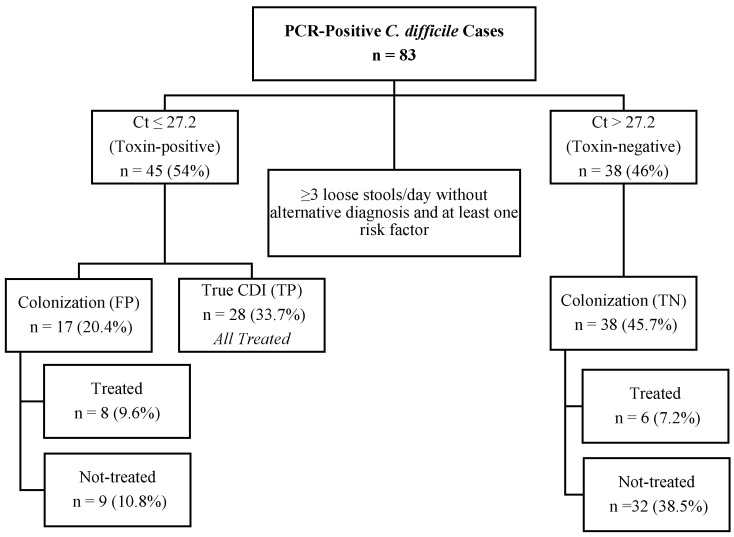
Flowchart of PCR-positive *C. difficile* pediatric cases (*n* = 83), classified by cycle threshold (Ct) values, clinical review determination, and treatment outcomes. CDI, *Clostridioides difficile* infection; Ct, Cycle threshold; FP, false positive; *n*, number; TN, true negative; TP, true positive.

Figure 1 shows a breakdown of the patients included in our study by PCR Ct value, clinical review determination, and treatment status. All 28 TP patients, 8 out of 17 FP patients, and 6 out of the true-negative (TN) patients were treated. The 28 TP patients met the clinical criteria for true CDI. In contrast, 14 treated patients classified as FP or TN were not confirmed as true CDI. Among these, 8 FP patients had documented co-infections with other enteric pathogens, while 6 TN patients received treatment based on clinical judgment and underlying risk factors, including inflammatory bowel disease (IBD) or active malignancy.

**Figure 2:**
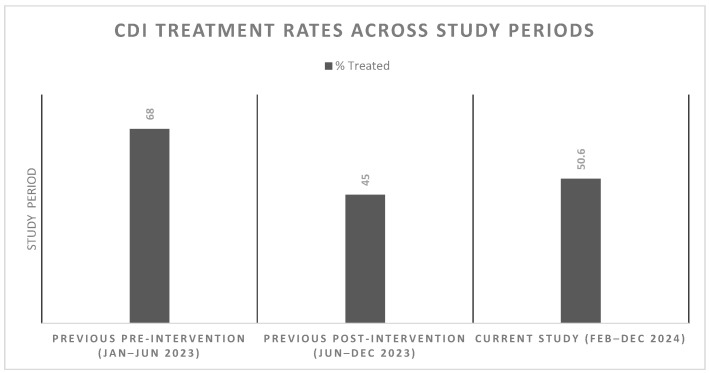
Bar chart showing CDI treatment rates across study periods: previous pre-intervention (Jan–Jun 2023), previous post-intervention (Jun–Dec 2023), and current study (Feb–Dec 2024). Percentages of treated patients are indicated for each period. Previous pre-intervention = PCR results reported only; previous post-intervention = PCR results + toxin results based on EIA + comment in EMR + phone consultation; current study = PCR results + toxin results based on Ct value + comment in EMR without phone consultation.

Our current results were compared with our previous study in which a clinical intervention was implemented to reduce unnecessary use of antibiotics for the treatment of CDI [12]. We observed a significant 17% decrease (*p* = 0.04) in the number of treated patients compared to the pre-intervention period in our previous study (68% treated) from 1 January to 24 June 2023. Conversely, there was a 5% insignificant increase (*p* = 0.60) in the number of treated patients relative to the post-intervention period in our previous study (45% treated) from 25 June 2023 to 16 December 2023. This difference between CDI treatment rates in our studies is illustrated in Figure 2.

## 4. Discussion

This follow-up study builds on our previous work demonstrating that a PCR Ct-guided diagnostic strategy can lower antibiotic consumption and improve antimicrobial stewardship in children with CDI [12]. Notably, our findings suggest that diagnostic accuracy and clinical decision-making for CDI in a population with high rates of asymptomatic colonization and co-infection with other enteropathogens can be further improved by combining PCR Ct values and clinical assessment [9,11].

In this study, a Ct cutoff of 27.2 was used to guide toxin-based reporting. Patients with Ct values below this threshold typically presented with ≥3 episodes of diarrhea and relevant risk factors, such as inflammatory bowel disease or underlying malignancy, and were treated with CDI-directed antibiotics, resulting in symptom resolution in most true-positive cases. In contrast, patients with higher Ct values more often exhibited milder or non-specific symptoms, and management decisions by physicians were guided by clinical assessment and laboratory results. Similarly, several studies have emphasized that interpreting Ct values in conjunction with clinical symptoms and patient risk factors supports more appropriate treatment decisions and helps limit unnecessary antibiotic exposure [9,10,11].

The QIAstat-Dx Gastrointestinal Panel (QGP) with a Ct cutoff of 27.2 showed excellent sensitivity and negative predictive value (both 100%) in all 28 patients who met the clinical definition of true CDI according to IDSA and SHEA, confirming it as a reliable rule-in test for CDI in children who are selected for testing based on appropriate clinical presentation and risk factors. Specificity was moderate at 69%, with a positive predictive value of 63%. These results are consistent with a previous pediatric study, which reported slightly lower specificity (65.9%) and positive predictive value (57.4%), suggesting that Ct-based reporting, combined with careful clinical interpretation, can improve diagnostic accuracy and reduce unnecessary treatment [13]. Although the Ct cutoff of 27.2 performed well in our cohort, it is assay- and platform-specific. Ct values can vary with extraction methods and PCR instruments; therefore, other laboratories should locally validate this Ct value cutoff before using [12].

Sixteen out of the 17 FP PCR tests were observed in patients co-infected with other enteropathogens, including pathogenic *E. coli*, *Campylobacter*, *Salmonella*, and *norovirus*. Determining the primary causative agent of acute gastroenteritis in patients with high prevalence of asymptomatic *C. difficile* colonization and multiple pathogens identified through PCR panels is challenging [14]. A high rate of co-detection of other enteropathogens, including *C. difficile*, has been documented across several multiplex platforms [14,15,16,17]. In this study, if *C. difficile* had not been classified as the main pathogen and excluded from reporting in 16 FP cases with co-infection, the specificity of the method would have increased to 97%. This finding underscores the importance of correlating clinical presentation and risk factors along with PCR results in the context of co-infections.

Our clinical review shows that 14 (17%) patients received unnecessary treatment for CDI (6 TN and 8 FP). The 6 TN patients were not documented to have 3 or more episodes of diarrhea in the EMR and/or had an alternative diagnosis, but they were still treated based on clinical assessment of the attending physicians and ongoing risk factors (IBD patients/oncology patients). Nevertheless, this also illustrates the complexity of interpreting *C. difficile* testing results in clinical practice in pediatric patients with risk factors such as IBD or malignancy undergoing chemotherapy. Previous studies have demonstrated that children with IBD or/and/or oncology patients are at increased risk for CDI, which may influence clinical decision-making [6,18].

The result of our study also shows that reporting toxin production results based on the PCR Ct value reduced the percentage of PCR-positive pediatric patients treated for CDI. Only 50.6% of PCR-positive tests led to CDI treatment, including 7.2% of patients with TN results, suggesting that our lab reporting protocol is effective in reducing CDI overtreatment. This study shows a significant 17% decrease (*p* = 0.04) in the number of pediatric patients treated for CDI compared to the pre-intervention period in our previous study [12]. Our results are consistent with Schwenk et al. [10], who found a 22% decrease in CDI treatment following Ct-based toxin reporting. The 5% increase in antibiotic use compared with the earlier post-intervention period may be attributed to phone consultations, which were not conducted during this study in comparison to the previous study. This highlights that while automated Ct-based reporting within the EMR can aid clinical decision-making, it cannot replace direct communication between clinicians and microbiologists. Thus, combining Ct-guided reporting with active clinical consultation is essential for reducing unnecessary treatments. Other studies have similarly found that EMR-based microbiology reporting alone may not effectively alter treatment decisions without direct consultation and interpretation by the microbiologist and clinician [19].

Limitations of our study include the small number of PCR-positive samples in our population, as the estimated prevalence of *C. difficile* in our pediatric population is 5%, low number of patients classified as true CDI according to our clinical review, and the lack of a third independent test to determine the cause of diarrhea in patients with co-infection, which may have overestimated false-positive results. The limited sample size also resulted in wide 95% confidence intervals for specificity and PPV, which reduced the statistical power of the diagnostic metrics. However, a comprehensive clinical review of patient symptoms and risk factors, along with a discussion with the direct caregivers, can help in minimizing this challenge to facilitate patient-centered decision-making [20,21].

## 5. Conclusions

The results of our study indicate that the use of NAAT assays as standalone tests for diagnosing CDI in children can be enhanced by using Ct-based reporting. Furthermore, our study suggests that a PCR Ct-based report combined with clinical presentation and risk factor assessment may help reduce unnecessary antibiotic use for CDI without compromising patient safety. Since the accuracy of molecular testing results may vary depending on the PCR assay used and the *C. difficile* prevalence in specific populations, PCR Ct values for CDI diagnosis should be validated in each particular setting.

## Figures and Tables

**Table 1:** Diagnostic performance of the QIAstat-Dx Gastrointestinal Panel for *Clostridioides difficile* infection at a cycle threshold (Ct) cutoff of 27.2, using clinical review as the reference standard.

Diagnostic Performance Metric	Values	Excluding 16 FP Patients with Co-Infection
TP	28	28
TN	38	38
FP	17	1
FN	0	0
Sensitivity	100% (88–100)	100% (88–100)
Specificity	69% (56–80)	97% (86–100)
PPV	63% (49–76)	97% (83–99)
NPV	100% (91–100)	100% (91–100)

FN, false negative; FP, false positive; TN, true negative; TP, true positive; NPV, negative predictive value; PPV, positive predictive value.

## Data Availability

The original contributions presented in the study are included in the article. Further inquiries can be directed to the corresponding author.
